# The Fate of Sulfamethazine in Sodium-Hypochlorite-Treated Drinking Water: Monitoring by LC-MS^*n*^-IT-TOF

**DOI:** 10.1155/2012/693903

**Published:** 2012-05-15

**Authors:** Tyler C. Melton, Stacy D. Brown

**Affiliations:** Department of Pharmaceutical Sciences, Bill Gatton College of Pharmacy, East Tennessee State University, Johnson City, TN 37614-1708, USA

## Abstract

Pharmaceutical compounds represent a rapidly emerging class of environmental contaminants. Such compounds were recently classified by the U.S. Geological Survey, including several antibiotics. An LC-MS/MS screening method for the top five antibiotics in drinking water was developed and validated using a Shimadzu LC-MS-IT-TOF. The separation was performed using a Waters Acquity UPLC BEH C18 column with a gradient elution. Sulfamethazine was exposed to conditions intended to mimic drinking water chlorination, and samples were collected and quenched with excess sodium sulfite. Kinetics of sulfamethazine degradation was followed as well as the formation of the major chlorinated byproduct (*m*/*z* 313). For the screening method, all five antibiotic peaks were baseline resolved within 5 minutes. Additionally, precision and accuracy of the screening method were less than 15%. Degradation of sulfamethazine upon exposure to drinking water chlorination occurred by first order kinetics with a half-life of 5.3 × 10^4^ min (approximately 37 days) with measurements starting 5 minutes after chlorination. Likewise, the formation of the major chlorinated product occurred by first order kinetics with a rate constant of 2.0 × 10^−2^. The proposed identification of the chlorinated product was 4-amino-(5-chloro-4,6-dimethyl-2-pyrimidinyl)-benzenesulfonamide (C_12_H_13_N_4_O_2_SCl) using MS^*n*^ spectra and databases searches of SciFinder and ChemSpider.

## 1. Introduction

For over thirty years, pharmaceutical compounds have been emerging as environmental contaminants, appearing in drinking and waste water all over the world. The persistence of pharmaceutical environmental contaminants, such as antibiotics, in the water supply is of increasing concern within the field of environmental toxicology. Several domestic and international entities have reported the presence of antibiotic compounds in ground water, municipal drinking water, and waste water. Some of these antibiotics include sulfonamides, fluoroquinolones, chloramphenicol, and trimethoprim [1–6]. A recent survey of 47 groundwater sites across 18 states conducted by the U.S. Geological Survey found the common veterinary and human antibiotic, sulfamethoxazole, to be present in 23% of the samples collected [7]. Similar results associated with high environmental levels of sulfamethoxazole were found in a survey of the Seine River in France [2] and the Guangzhou section of the Major Pearl River, China [3]. While the absolute concentration of these drugs in drinking water is certainly subclinical, their presence has the potential to contribute to the emergence of antibiotic-resistant bacterial strains. Bacterial strains that are resistant to one or several commercially available antibiotic drugs have been detected in surface, waste, and drinking water supplies, yet the link between this phenomenon and antibiotics in drinking water is unknown [8–11].

An added layer of complexity in this problem lies in the potential biotransformation antibiotic compounds can undergo during drinking water chlorination. Chlorination, typically in the form of sodium hypochlorite, is a common mechanism of drinking water disinfection used in the United States. In general, drinking water sterilization byproducts have been linked to adverse reproductive effects, certain types of cancer, and developmental problems [12]. The chemicals used in drinking water chlorination are in high enough concentrations to initiate chemical reactions with low levels of organic compounds within water, with antibiotics not being exempt from such chemistry. Chlorination has been shown to reduce the concentration of certain parent drugs in drinking water [13]. The chlorination processes have also been shown to generate chlorinated versions of ethynylestradiol [14] and have been associated with the formation of multichlorinated and hydroxylated forms of trimethoprim [15]. The effect of chlorination has been studied for several nonantibiotic drugs, including oxcarbazine [16], gemfibrozil [17], acetaminophen [18], atenolol [19], fluoxetine [20], and metoprolol [21]. However, these studies are few in comparison to the variety of pharmaceutical contaminants our environment faces.

A long-term goal of this research is to understand the chemical fate of antibiotics in the water supply when they are exposed to chlorination in the drinking water treatment process and the subsequent contribution these derivatives have to antimicrobial drug resistance. It is important to know if the active antibiotic moiety is retained. To this end, we have developed a screening method for accurate and precise quantification of the top five most common antibiotic contaminants in US waters, including sulfamethazine, sulfamethoxazole, trimethoprim, lincomycin, and enrofloxacin. Additionally, we have applied IT-TOF accurate mass capabilities coupled with molecular formulae search engines to structurally characterize the chlorinated byproduct of sulfamethazine. No other published method currently exists to monitor all five of these antibiotics in drinking water, nor has anyone previously characterized the chlorination fate of sulfamethazine.

## 2. Methods

### 2.1. Materials and Equipment

All drug standards (sulfamethoxazole, sulfamethazine, lincomycin, enrofloxacin, and trimethoprim) were of analytical grade and were acquired from Sigma-Aldrich (St. Louis, MO). Additionally, the chemicals used in the chlorination experiments, sodium hypochlorite and sodium sulfate, were of analytical grade and also purchased from Sigma-Aldrich. The free chlorine concentration in the samples and stock solutions was monitored using a Hach Pocket Colorimeter II (Loveland, CO). The solvents used included methanol, water, and 0.1% v/v formic acid in acetonitrile. All of these solvents were of LC-MS grade (Burdick & Jackson, Morristown, NJ). The HPLC column was a Waters Acquity UPLC BEH C18 column, 1.7 micron, 2.1 × 100 mm (Milford, MA). The Shimadzu liquid chromatography system consisted of two LC-20AD pumps with UFLC-XR upgrade, SIL-20ACHT auto sampler, CTO-20A column oven, DGU-20A_3_ degasser, and CBM-20A Communications module. This system was coupled to the Shimadzu IT-TOF mass spectrometer with an electrospray (ESI) source (Columbia, MD). The mass spectrometer utilized UHP nitrogen as a nebulizing and drying gas and UHP argon as a collision gas, both supplied by Airgas (Johnson City, TN). Molecular formulae searches were performed using the open-access ChemSpider (http://www.chemspider.com), coordinated by the Royal Society of Chemistry in Cambridge and SciFinder, a subscription-based service of the American Chemical Society.

### 2.2. LC-MS/MS Conditions and Screening Method

The LC-MS/MS run included a gradient elution using 80% water/20% acetonitrile with 0.1% formic ramped to 70% organic over 7 minutes at a flow rate of 0.200 mL/min. Data was acquired in automatic MS/MS using positive ion electrospray (ESI) mode with a CDL and heat block temperature of 200°C. Liquid nitrogen, used as the drying and nebulizing gas, was kept at a flow of 1.5 L/min. Argon was used as the collision gas for the MS^*n*^ experiments. Drug solutions (including sulfamethoxazole, sulfamethazine, lincomycin, enrofloxacin, and trimethoprim) for the method development and precision/accuracy experiments were prepared in ion-free water in the following concentrations (*n* = 3 at each concentration for each day): 10 *μ*g/mL, 7.5 *μ*g/mL, 5 *μ*g/mL, 2.5 *μ*g/mL, 1.0 *μ*g/mL, 0.75 *μ*g/mL, 0.5 *μ*g/mL, 0.25 *μ*g/mL, 0.1 *μ*g/mL, and 0.05 *μ*g/mL. These dilutions were prepared from 1 mg/mL and 100 *μ*g/mL stock solutions in 50/50 methanol water. Following each LC-MS/MS run, the data was collected using extracted ion chromatograms from MS^1^ data on the molecular ions for each drug. For calibration purposes and assessment of method precision, the peak areas were determined using the Shimadzu Quant Browser software in Postrun mode. Calibration curves and replicates at each concentration were run over three days (*n* = 3 at each concentration for each day).

### 2.3. Kinetics Experiment

Chlorination experiments mimicked common water treatment practices with a chlorine concentration of 2 mg/L. Ten *μ*g/mL sulfamethazine standards were exposed to 380 *μ*mol/mL free absolute chlorine (FAC) achieved by dilution of 5% sodium hypochlorite reagent and verified using a Hach Pocket Colorimeter II. Triplicate aliquots were prepared in amber and clear vials. One mL samples were removed from the vials at various time points (5 min, 10 min, 15 min, 20 min, 30 min, 60 min, 2 hr, 3 hr, 4 hr, 6 hr, 8 hr, 24 hr) and the progress of the reactions halted with the addition of excess sodium sulfite: Na_2_SO_3_ + Cl_2_→ 2NaCl + SO_3_^2^^−^ [22]. Upon quenching, the samples were filtered using a 0.22 micron syringe filter and immediately analyzed using the LC-MS/MS conditions stated above. Peak area of the sulfamethazine peak in each sample was integrated and used to determine the reaction kinetics of the drug degradation under these chlorinated conditions. The other peaks in the chromatogram were explored for the chlorine isotope pattern, and the major chlorinated product (*m*/*z* 313) was chosen for additional monitoring.

### 2.4. MS^*n*^ Experiment

The major chlorinated product from sulfamethazine's reaction with sodium hypochlorite, m/z 313, was subjected to MS^5^ in order to propose a structure for this derivative; however, no further fragmentation was noted after MS^4^. Initially, a full scan MS^1^ spectrum was used to identify relevant fragments, and subsequent MS^*n*^ fragmentations were proposed in the following manner: MS/MS of *m*/*z* 313; MS^3^ of *m*/*z* 313,279; MS^4^ and MS^5^ of *m*/*z* 313, 279, 186. Prior to MS^*n*^ experiments, the instrument was calibrated with sodium trifluoroacetic acid in acetonitrile, as recommended by the manufacturer. At each MS^*n*^ level, the formula predictor software was used to generate possible formulae for the fragments, and knowing the fragmentation pattern for the parent drug, a proposed structure was generated from the MS^*n*^ pattern. The molecular formula of this derivative was searched on ChemSpider and SciFinder, and a match, which posed significant similarity to the parent sulfamethazine structure, was generated. The MS^*n*^ experiment was repeated over three different days, following instrument calibration each day, to ensure that a consistent fragmentation pattern and subsequent derivative structure was generated.

## 3. Results and Discussion

### 3.1. Screening Method Precision and Accuracy

**Figure 1:**
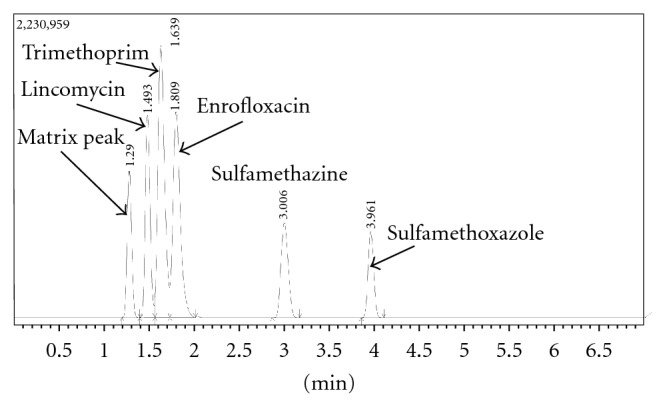
Chromatogram from antibiotic screening method, concentration of each compound = 5 *μ*g/mL.

All five antibiotic peaks were well resolved within 5 minutes, as shown in Figure 1 where each compound is represented by a 5 *μ*g/mL concentration. Additionally, precision (represented by % relative standard deviation) and accuracy (represented by % error) is acceptable (<15%) for each drug. Due to variable sensitivities, the limits of detection (as defined as 3 : 1 signal to noise ratio) were different for each compound, ranging from 0.1 *μ*g/mL for trimethoprim to 1 *μ*g/mL for sulfamethazine and sulfamethoxazole. These data are summarized in Table 1. The repeatability and accuracy of this assay conferred confidence that it would be useful for monitoring drug degradation under chlorinated conditions. The calibration slopes were also reproducible, with % RSD ranging from 3.1–11.5% over three days (data not shown). Although increased sensitivity could have been achieved by conducting direct MS/MS experiments on the relevant *m*/*z* for each drug, the full scan nature of this assay is preferred because it does not exclude additional peaks that are likely to appear as a result of chlorination treatment. Trimethoprim and sulfamethoxazole have already been monitored to some degree following chlorination treatment [15, 21]; however, they were included in the screening assay because they are in the top five antibiotic contaminants in the USGS survey [6, 7]. Because all of the top five antibiotic contaminants are included in this assay, the method can be used for monitoring any of these individually as well as any combination thereof.

### 3.2. Kinetics of Sulfamethazine Degradation

**Figure 2:**
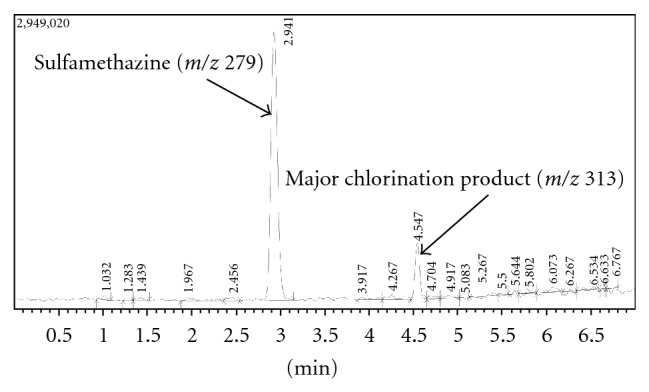
Postchlorination chromatogram, sulfamethazine (10 minute reaction time), showing major chlorination product (*m*/*z* 313).

**Figure 3:**
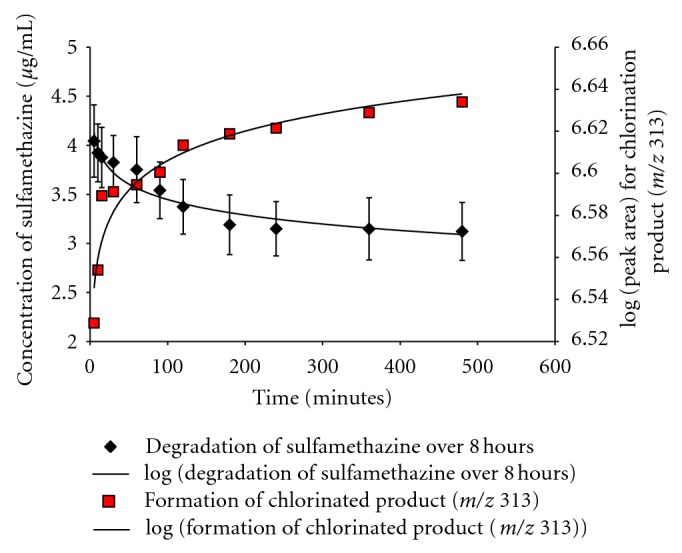
Formation of major sulfamethazine product following chlorination treatment over 8 hours.

The kinetics of sulfamethazine chlorination was followed as well as the formation of the major chlorinated byproduct (*m*/*z* 313). This post-chlorination of sulfamethazine, following a 10 minute reaction with NaClO, is shown in Figure 2. The major chlorinated product, which was identified as chlorinated by the mass spectral isotope pattern on the molecular ion, elutes at 4.5 minutes. Degradation of sulfamethazine upon exposure to drinking water chlorination occurred by first order kinetics with a half-life of 5.3 × 10^4^ min (approximately 37 days) with measurements starting 5 minutes after chlorination. Likewise, the formation of the major chlorinated product occurred by first order kinetics with a rate constant of 2.0 × 10^−2^. These data are shown in Figure 3, out to a reaction time of 480 minutes. Data after 8 hr did not demonstrate significant additional sulfamethazine degradation, as demonstrated by their lack of statistically significant difference from the 480 minute time point. Consequently, to show the detail of the reaction approaching steady state, the data are only shown through the first 8 hours. The data shown are from the amber-vial experiments; we saw no significant difference in kinetics between these conditions versus the clear vials, indicating minimal to no contribution of UV light to the reaction progression during the time monitored.

### 3.3. MS^*n*^ of Major Product

**Figure 4:**
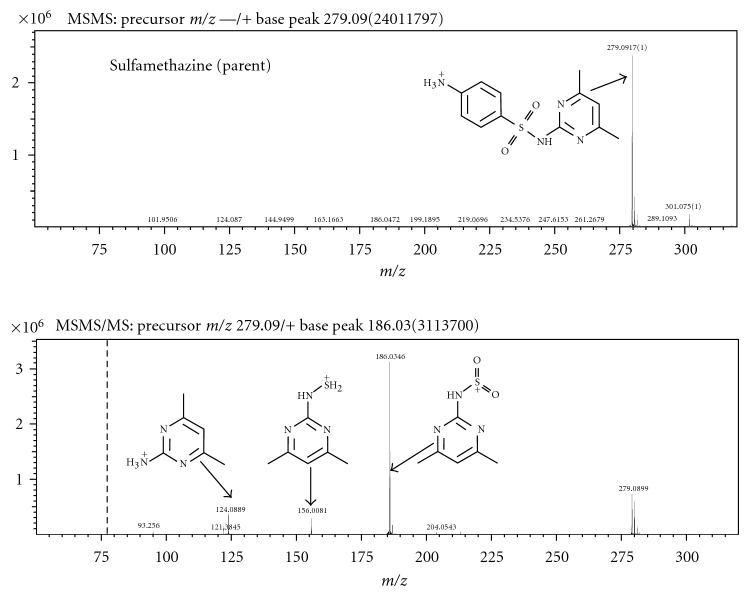
MS/MS and MS^3^ spectra of sulfamethazine parent with structure and proposed fragmentation pattern.

**Figure 5:**
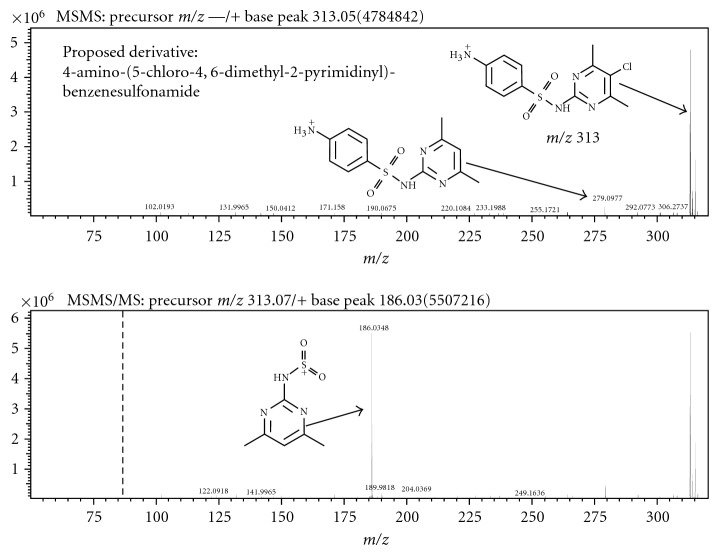
MS/MS and MS^3^ spectra of chlorinated sulfamethazine derivative with structure and proposed fragmentation pattern.

The MS/MS and MS^3^ spectra for sulfamethazine are shown in Figure 4. The similarities between these spectra and that of the derivative are striking (Figure 5), especially the major fragment in MS^3^ of *m*/*z* 186. Additionally, the MS^4^ spectrum for the chlorinated derivative shows a weak *m*/*z* 124, which appears in the MS^2^ spectrum of sulfamethazine. Finally, the MS/MS spectrum of the derivative shows an *m*/*z* 279 fragment, which corresponds to the loss of chlorine from the parent molecule and also matched the *m*/*z* of the parent, indicating the addition of only one chlorine atom upon hypochlorite exposure to the parent. The one chlorine addition to the parent is confirmed in the isotope pattern of the *m*/*z* 313 peak, which shows a one-third size *m*/*z* 315 fragment. This characteristic chlorine isotope pattern is not seen in any of the fragment spectra from the derivative, confirming that loss of chlorine is the first process to occur upon fragmentation. Based on MS^*n*^ data, molecular formulae generated by the Formula Predictor software (Shimadzu), and results from the SciFinder and ChemSpider databases, the proposed identification of the chlorinated product is 4-amino-(5-chloro-4,6-dimethyl-2-pyrimidinyl)-benzenesulfonamide (C_12_H_13_N_4_O_2_SCl). This compound was previously synthesized by Drake et al. by coupling N-acetylsulfanilyl chloride to the amine in pyridine followed by hydrolysis with sodium hydroxide [23]. This reaction involved a 2-hour incubation time at 60–70°C temperatures to achieve a 47% yield. Based on the relative peak areas, our yields range from 8.8–7.1% over a 24-hour reaction time.

## 4. Conclusions

 This method demonstrates an accurate and precise way to screen for the top five antibiotic contaminants in drinking water as identified by the USGS. Being able to track the fate of these compounds in drinking water, especially following chemical treatment, allows for approximation of drug half-life in these conditions, helping us understand their persistence in the environment. Furthermore, the use of hybrid ion-trap time-of-flight mass spectrometry enables high mass accuracy MS^*n*^ experiments to be conducted on the chlorinated antibiotic derivatives in conjunction with quantitative determination of the parent compounds. Historically, drinking water chlorination has been shown to produce derivatives of other organic compounds, termed chlorination byproducts, and these experiments demonstrate that sulfonamide antibiotics are no exception to this phenomenon. Currently, the persistence of the 4-amino-(5-chloro-4,6-dimethyl-2-pyrimidinyl)-benzenesulfonamide in drinking water is unknown, but if it follows the trend of other environmental halogenated organics, the persistence is possibly quite high. This has implications for the contribution that these chlorinated antibiotics in the environment may have toward promoting bacterial resistance. Finally, this work demonstrates that the chlorinated derivative of sulfamethazine can be synthesized, albeit with much lower yields, under conditions much simpler than previously reported.

## Figures and Tables

**Table 1:** LC-MS precision, accuracy, calibration ranges, and limits of detection for each compound encompassed in the screening method (*n* = 3 for each concentration for each day for each drug).

Drug	Calibration	Interday precision	Inter-day accuracy	Limit of detection
	Range (*μ*g/mL)	(% RSD) Range	(% error) Range	(LOD)

*Lincomycin *(406.5 g/mol)	10–0.75	3.7–13.3%	0.30–14.8%	0.5 *μ*g/mL
*Trimethoprim *(290.3 g/mol)	10–0.5	3.8–6.3%	0.40–14.8%	0.1 *μ*g/mL
*Enrofloxacin *(359.4 g/mol)	10–1.0	3.9–13.8%	1.2–14.6%	0.75 *μ*g/mL
*Sulfamethazine *(278.3 g/mol)	10–2.5	4.2–12.6%	0.95–12.7%	1 *μ*g/mL
*Sulfamethoxazole *(253.3 g/mol)	10–2.5	2.1–7.2%	0.23–14.7%	1 *μ*g/mL
